# The surviving sepsis campaign: fluid resuscitation and vasopressor therapy research priorities in adult patients

**DOI:** 10.1186/s40635-021-00369-9

**Published:** 2021-03-01

**Authors:** Ishaq Lat, Craig M. Coopersmith, Daniel De Backer, Craig M. Coopersmith, for the Research Committee of the Surviving Sepsis Campaign, Daniel De Backer, Clifford S. Deutschman, Laura Evans, Ricard Ferrer-Roca, Judith Hellman, Sameer Jog, Jozef Kesecioglu, Ishaq Lat, Flavia Machado, Greg Martin, Ignacio Martin-Loeches, Mark E. Nunnally, Andrew Rhodes

**Affiliations:** 1 Department of Pharmacy, Shirley Ryan Abilitylab, Chicago, IL USA; 2 Department of Surgery and Emory Critical Care Center, Emory University, Atlanta, GA USA; 3 Department of Intensive Care, Chirec Hospitals, Université Libre de Bruxelles, Brussels, Belgium

**Keywords:** Fluid resuscitation, Sepsis, Septic shock, Vasoactive agents, Vasopressor

## Abstract

**Objective:**

To expand upon the priorities of fluid resuscitation and vasopressor therapy research priorities identified by a group of experts assigned by the Society of Critical Care Medicine and the European Society of Intensive Care Medicine.

**Data Sources:**

Original paper and literature search.

**Study Selection:**

Several members of the original task force with expertise specific to the area of fluid resuscitation and vasopressor therapy.

**Data Extraction:**

None.

**Data Synthesis:**

None.

**Conclusion:**

In the second of a series of manuscripts subsequent to the original paper, members with expertise in the subjects expound upon the three identified priorities related to fluid resuscitation and vasopressor therapies. This analysis summarizes what is known and what were identified as ongoing and future research.

## Introduction

The Surviving Sepsis Campaign (SSC) is a long-standing initiative of Society of Critical Care Medicine and European Society of Intensive Care Medicine designed to improve mortality from sepsis. The Campaign has released four sets of guidelines [1–4], with another due to be published in 2021. Although recommendations within the SSC guidelines have been associated with improved outcomes [5–7], the guidelines are often unable to make more specific recommendations in multiple areas of clinical importance due to ongoing gaps in the literature.

In an attempt to define priorities for research within the field of sepsis, SSC created a research committee that was charged with developing a list of research questions related to sepsis. This led to the joint publication of “Surviving Sepsis Campaign Research Priorities for Sepsis and Septic Shock” in *Critical Care Medicine* and *Intensive Care Medicine* in August 2018 [8, 9]. The initial document presented a broad overview, identifying 26 questions to explore as research priorities in several domains with an explicit intention to publish separate papers with more detailed descriptions for each domain in the future. This article is the second in a series of manuscripts (following a prior effort devoted to basic science research), which will expand upon the three research questions related to fluid resuscitation and vasopressors originating from the broader publication.

## Methods

The content of the initial research priorities article was developed by asking each committee member to identify the research questions they believed were most important. Using a modified Delphi approach as outlined in the original article, the Task Force members focused on the original 88 suggestions to a series of 26 questions focused on all domains related to sepsis. These included questions related to clinical management (as in the SSC guidelines) and domains that were outside the scope of the guidelines (basic science, scoring, epidemiology, long-term outcomes, etc.). These top research priorities were presented in the original publication [8, 9]. The 26 questions were grouped by thematic similarity, with the plan to expand each question to a level of detail precluded by space constraints in the original publication. From this list of 26 questions, three committee members with specific expertise in the domain of fluid resuscitation and vasopressor therapies were tasked with generating expanded reviews of the three questions on fluid resuscitation and vasopressors generated in the original article for the treatment of septic shock. Consistent with existing definitions of septic shock, the article focuses on the subset of sepsis where underlying cellular and circulatory abnormalities are substantially enough to increase mortality. The final list of questions was broad and comprehensive in an attempt to add to the collective body of knowledge regarding therapeutic benefit in addition to describing mortality. These in-depth reviews were summarized and edited by the group as a whole.

## Overview of the presentation

The three fluid and vasopressor questions identified by the task force as a whole are as follows:

1. What are ideal endpoints for volume resuscitation and how should volume resuscitation be titrated?
2. What is the optimal fluid for sepsis resuscitation?
3. What is the optimal approach to selection, dose titration, and escalation of vasopressor therapy?

The format for each of the three questions directly mirrors that used in the previously published overview, which contains a more extensive description of the methods [8, 9]. One question was assigned to each author for review and summary. The presentation of each question is followed by a critique of the existing evidence of what is currently known of the subject. Subsequently, each author presented what is not known regarding each question and the gaps in our current understanding of the subject. For each question, the suggested research questions are iterative and aim to address broad areas of uncertainty. Accordingly, clinical outcomes may need to be refined to not only determine mortality benefit, but also inform stakeholders on relevant outcomes of interest, such as, quality of life, organ function, and resource utilization. Finally, each author concluded their overarching question by presenting a proposed list of research questions deemed worthy of further inquiry. This list of questions was purposefully intended to be exhaustive, and the authors acknowledge that they may not be answered in the coming decade, but are necessary to describe so as to provide a roadmap for where research efforts should be directed.

### Question 1: what are ideal endpoints for volume resuscitation and how should volume resuscitation be titrated?

#### What is known

The rationale for fluid resuscitation is largely derived from multiple experimental and clinical studies [10–15]. Fluid resuscitation increases cardiac output, at least at the early stages of sepsis [13, 16]. In addition, fluid resuscitation increases microvascular perfusion in patients with septic shock [17], and this is associated with improved organ function [18].

The impact of fluid resuscitation on outcome is less obvious and is mostly supported by experimental data. Large-scale interventional trials investigating goal-directed therapy did not directly address the amount, timing, or guiding clinical variables beyond central venous oxygenation (Scvo_2_). In preclinical models of sepsis, fluid administration prolongs survival time compared with no fluid resuscitation [11], even though long-term outcomes could not be evaluated from these types of studies. Specifically, out-of-hospital fluid administration during transport by paramedics is associated with an improved outcome in hypotensive patients [19], it has not been associated with a benefit, and it may even be associated with increased mortality, in normotensive patients [20].

Although early fluid administration is beneficial to delayed fluid administration, the optimal amount of fluid required for an individual patient varies. “Optimal” would infer the quantity of fluid administered that restores perfusion to end organs while not worsening end-organ function. The Hour-1 bundles recommend initiating 30 mL/kg of IV crystalloid to patients with suspected sepsis within 1 h for hypotension or lactate level greater than or equal to 4 µmol/L [21]. Although this is a strong recommendation, it is based upon a low quality of evidence, where the 30 mL/kg dose is derived from a statistical correlation between mortality and amount of fluid administered [22]. Therefore, determining the optimal amount of fluid to be administered remains a critical issue through continued inquiry. Analysis of large databases suggest that there may be a U-shaped response curve, where limited amounts as well as large amounts of fluid administration are associated with worse outcomes. The best response was observed when fluid resuscitation was administered in volumes between 15 and 45 mL/kg [22], although this range is too wide to be applied readily in clinical practice. Further, data from over 50,000 patients demonstrated that delay in fluid administration was not associated with an increased risk of death [6]. One potential explanation is “time zero” in these studies was defined as presentation to hospital and not time of sepsis recognition. Further, the benefits of fluid resuscitation may be dependent on the severity of illness in the continuum of sepsis and underlying comorbidities [23–25].

Shortly after administration, most patients respond to fluids in the form of improved blood pressure, but this proportion decreases rapidly over time [26]. After initial fluid bolusing, the amount of fluid required to maintain goal blood pressure varies between patients based upon a number of factors, ranging from severity of dehydration to ongoing fluid losses to severity of illness to underlying comorbidities. Understanding when to continue volume loading and when to discontinue additional fluid administration is of critical importance, since excessive fluid administration appears to be detrimental, as suggested by the association between positive fluid balance and poor outcome in observational trials [27, 28]. A meta-analysis of nine randomized trials comparing “low” versus “high” volumes of resuscitation found no difference in outcome [29], suggesting that individualization of fluid therapy is desired. The duration of the hemodynamic effects of fluids is determined by the amount of fluid administered, capillary leak, and hemodynamic adjustments (including volume adjustments between stressed and nonstressed volumes) resulting from the resolution of compensatory mechanisms initiated during hypovolemia. Very few studies have evaluated the impact of the duration of the hemodynamic effects of fluids in critically ill patients, and it seems that these effects may last less than 2 h [30]. Conceptually, fluid administration improves cardiac output and tissue perfusion. However, it is critical to note that fluid administration yields inconsistent effects in a cohort of patients. As such, there is increasing recognition of the need for individualizing care. Resuscitation by formula is not suitable for achieving endpoints of resuscitation across a heterogeneous group of patients, as varied needs would result in some patients receiving too much and others not enough fluids. Further, although the rationale for initiating fluid resuscitation is the correction of tissue malperfusion through an improvement in cardiac output, in practice, fluids are often administered in order to correct hypotension or oliguria, whereas indices assessing tissue perfusion, including cardiac output, are often not readily assessable [31].

No single measurement best identifies how an individual patient will respond to fluid administration. Historically, static indices such as intravascular pressures (central venous pressure [CVP] and pulmonary artery occlusion pressure) and cardiac volumes (by echocardiography or transpulmonary thermodilution) were used to guide fluid administration. However, the predictive power of these variables is moderate, with extreme values correctly predicting clinical response to fluid resuscitation but leaving a large gray zone in between [32]. Targeting specific CVP values may be valuable when other, more reliable, variables predicting fluid responsiveness are not applicable or available. However, when more reliable measures are available, CVP is more useful in gauging the potential risk of further fluid administration rather than an accurate predictor of fluid responsiveness [33]. In contrast, dynamic variables such as inferior vena cava compression on bedside ultrasound, pulse pressure variation, and passive leg raise are more reliable in predicting the increase in cardiac output in response to fluid administration. Even though the physiologic basis of using dynamic rather than static indices of fluid responsiveness is sound, proof that using these indices improves outcome is still lacking. Although a systematic review of dynamic versus static indices of preload demonstrates the superiority of dynamic variables to predict the response to fluids, this failed to demonstrate a difference in outcome [34]. As such, the SSC suggests using dynamic variables over static variables for predicting fluid responsiveness. Unfortunately, numerous prerequisites are needed to make dynamic measurements valid, which challenge feasibility. Several additional indices can also be considered in determining optimal fluid resuscitation, including Scvo_2_, lactate, venoarterial Pco_2_ gradients, and capillary refill time (CRT). Notably, in the ANDROMEDA-SCHOCK trial, patients randomized to CRT-guided resuscitation experienced a decrease in the risk of death compared with patients randomized to lactate-guided therapy [26, 35]. Importantly, all patients included in the ANDROMEDA trial had persistently elevated lactate levels after initial fluid resuscitation, indicating the likelihood that some patients in the lactate group unduly received additional fluid resuscitation even though tissue perfusion may have already normalized at time of assessment. Since this trial did not investigate CRT in isolation of an elevated lactate, the efficacy of applying fluid resuscitation in patients with prolonged CRT paired with normal lactate levels is unknown. The benefit to lactate-guided therapy may be in the recognition of hypoperfusion sooner in normotensive patients and otherwise considered to not experiencing shock. The results of the ANDROMEDA trial suggest that for those patients who are hypotensive, hyperlactatemic, and have delayed CRT, a clinical evaluation of CRT assessment is superior to pursuing a biomarker-guided strategy. Similarly, pursuing a lactate-guided strategy may be beneficial in the earliest stages of shock, where hyperlactatemia is more indicative of hypoperfusion as opposed to the progressed stages of shock, where hyperlactatemia is more indicative of decreased clearance.

The concept of fluid restriction has also been evaluated in limited trials. One of the most relevant studies tested fluid resuscitation according to two different levels of triggering physiologic variables, with the restrictive arm requiring more severe alterations prior to fluid administration [36]. An important limitation of the trial was that fluids were administered without testing fluid responsiveness, resulting in extra fluid administration in both groups. The lack of a testing procedure to determine fluid responsiveness would be perpetuated in clinical practice until a decision is made to abort fluid administration for lack of desired treatment effect or physiologic worsening. In addition, some have proposed that early vasopressor administration in sepsis may help to limit fluid resuscitation by counteracting the sepsis-induced venous dilation. Indeed, venous dilation increases the unstressed volume at the expense of stressed volume, and vasopressors may help to redistribute volumes by constricting capacitance veins [37]. In experimental sepsis, early norepinephrine administration combined with fluid resuscitation decreased volume requirements while resulting in similar tissue perfusion as later norepinephrine administration [38]. Unfortunately, human data testing the interactions between vasopressors and fluid therapy in a prospective setting are limited. In a small series of septic patients, norepinephrine administration increased cardiac output by increasing mean systemic pressure (reflecting redistribution of blood from unstressed to stressed volume) [39] and cardiac preload indices [40].

#### What is not known—gaps in our understanding—directions for future research

1. Should we individualize the initial amount of fluid resuscitation and, if so, how? Applying a standard fluid dose to all patients with sepsis is inconsistent with other areas of sepsis management, where efforts are made to personalize care where possible. What alternative strategies can be readily used in clinical practice to individualize the initial amount of fluid resuscitation to maximize benefit and reduce harms?
2. Which variables should be used to titrate fluid resuscitation? Rigorous trials comparing various dynamic and static variables to evaluate fluid responsiveness are needed. Furthermore, testing various dynamic variables in subgroups of patients with sepsis would be informative. Trials should be designed to incorporate multiple methods of assessing fluid responsiveness.
3. What is the optimal timing for fluid resuscitation? After rapid identification of sepsis and septic shock, time-series studies of fluid resuscitation are needed, especially evaluating the benefits and harms of additional therapies (e.g., vasopressors) and their respective interplay.
4. Which variables should be used to trigger fluid resuscitation and deresuscitation? Related to the timing of fluid resuscitation, should a particular set of physiologic variables be assessed in a serial fashion to determine when to shift from resuscitation to deresuscitation in a structured manner?
5. Is a combined approach using several indices of fluid responsiveness better than using a single measurement in isolation? Furthermore, how should various measures be prioritized to inform clinical judgment?
6. What is the efficacy of using CRT to guide resuscitation in patients with normal lactate levels? Would the use of a rapidly assessable physiologic test be of value in patients without a derangement in lactate levels?
7. What is the duration of effect of fluid resuscitation? Recognizing that the duration of benefit of fluid resuscitation will vary between patients, having some population estimates in mind via pharmacokinetic studies would be useful to clinicians in evaluating therapeutic options and subsequent care.
8. Do early vasopressors limit the amount of fluids required to reach the same hemodynamic target? If so, what is the appropriate ratio of fluids and vasopressors? Identifying if there is a benefit to applying a polytherapeutic approach to early resuscitation is particularly interesting given the concerns regarding excessive fluid administration. A polytherapeutic approach of early fluid administration and vasopressors using a standardized assessment approach considerate of patient variables may be more precise and individualized, thereby maximizing benefit and limiting adverse effects.
9. Should the initial resuscitation of fluids be restricted to hypotensive patients or patients with lactate greater than or equal to 4 mmol/L? In an effort to limit the effects of excessive fluid administration while maximizing benefit in those patients with malperfusion, comparing strategies of fluid resuscitation in distinctive groups of septic shock would be useful.
10. Should the initial fluid resuscitation be administered as a fixed dose of 30 mL/kg or should it be administered in smaller aliquots, with further administration contingent on reassessing the patient condition prior to administering the next bolus? Although the 30 mL/kg bolus carries the risk of under- and overdosing, it has the advantage of being relatively simple to apply, especially in resource-limited settings where advanced measurements cannot be obtained. Administration of smaller doses, repeated as needed according to hemodynamic assessment, has the advantage of fine-tuning fluid administration according to an individual patient’s needs. The risk associated with this approach is the potential for adding additional complexity, while still resulting with relatively similar amount of fluids administered and diverting the healthcare team from completing other essential tasks in the initial management of the patient with sepsis.
11. Should sicker patients receive a greater amount of initial resuscitation? In the continuum of sepsis and septic shock, should those patients with more severe physiologic derangements receive greater initial amounts of fluid resuscitation to restore perfusion at the cellular level? Understanding that there may be dose gradient to the initial volume of fluid resuscitation based on the severity of shock would be informative and guide further, targeted research in the field.
12. Should resuscitation differ in patient populations less able to tolerate large volumes? In an effort to individualize sepsis care for subgroups at greater risk of harm from vigorous resuscitation, trials evaluating subgroups of patients with particular comorbidities (heart failure, dysrhythmias, end-stage renal disease) are necessary. The ideal trial in this domain should test two fluid resuscitation strategies using tiered severity in the triggering variables, whereas the fluid boluses should be of similar amount and given only after the evaluation of fluid responsiveness. Ideally, the study should be stratified for subgroups such as heart failure and kidney disease.
13. What is the optimal strategy for fluid resuscitation in resource-limited settings? Data on resuscitation vary significantly between well-resourced and resource-limited settings [41], as in sub-Saharan Africa, the administration of fluids was associated with an increased risk of death. Understanding the physiology of fluid resuscitation in the absence of other elements of critical care provided in ICUs with higher resources is a key question for a large portion of the world’s population. Developing simplified strategies for patient assessment and fluid administration would greatly improve the quality of care delivered.

### Question 2: what is the optimal fluid for sepsis resuscitation?

#### What is known

The current SSC guidelines recommend crystalloids as the fluid of choice for initial resuscitation and subsequent intravascular volume replacement in patients with sepsis and septic shock (Table 1) [42]. The guidelines also recommend using either buffered solutions or saline for resuscitation, based upon an absence of published data demonstrating a difference in survival [42]. However, since the publication of the SSC guidelines, the SMART trial reported a decreased occurrence of the composite endpoint of major adverse kidney events, mortality, need for new renal replacement therapy, or persistent renal dysfunction in critically ill patients who received buffered solutions (lactated Ringer’s or acetated/gluconated buffered solution) compared with saline through 30 days or hospital discharge [43]. The effect in this landmark trial was modest, preventing new renal replacement therapy initiation, persistent renal dysfunction, or death in one of every 94 ICU patients. Although the trial enrolled nearly 16,000 patients, the ICUs and hospital allocation were limited. Importantly, the biggest difference in the composite outcome was noted in septic patients and in those receiving larger amounts of volume resuscitation, suggesting the benefits may be greater in these patient populations. Additionally, a subset analysis of the SMART trial looking only at septic patients suggests a mortality benefit in patients treated with balanced crystalloid solutions [44]. However, the generalizability of this trial is unclear since it was a single-center, unblinded trial. The SPLIT trial enrolled 2,278 patients in a cluster randomized controlled trial of four ICUs in New Zealand comparing saline or acetated/gluconated buffered solution [45]. This trial found no difference in the 90-day rate of acute kidney injury (AKI) between treatment groups (point estimate, 0.4%; relative risk [RR], 1.04; *p* = 0.77), although the study included a majority of elective postoperative patients with a lower risk of AKI compared with medically ill patients.

**Table 1:** Composition of commonly used isotonic crystalloid solutions

Fluid	pH	Na^+^ (mmol/L)	Cl^–^ (mmol/L)	K^+^ (mmol/L)	Ca^2+^ (mmol/L)	Mg^2+^ (mmol/L)	Other
Plasma	7.4	180	100	5	2.2	1	
Sodium chloride 0.9%	5.4	154	154	–	–	–	
Lactated Ringer’s	6.5	131	111	5.4	2	–	Lactate 28
Plasma-Lyte 148	5.5	140	98	5	–	2.5	Acetate 27
							Gluconate 23

Dash denotes absent from the fluid

A similar but smaller effect was found when comparing saline with buffered solutions in the same center for noncritically ill patients admitted from the emergency department to the hospital wards [46]. When looking at individual patient-centric outcomes including mortality and AKI (rather than a composite outcome), recent meta-analyses are conflicting as to whether balanced crystalloid solutions are superior to saline, both in critical illness in general and in sepsis specifically [47–49]. Of note, trials comparing buffered solutions with saline have generally not considered arterial blood gases and laboratory data in their design. One may potentially criticize the continued administration of saline in a patient with hyperchloremic acidosis, as this deviates from the current practice of many clinicians [50]. Similarly, retrospective or database studies have reported consistent effects of reduced incidence of in-hospital mortality, AKI, and need for renal replacement therapy [51].

A recent systematic analysis demonstrated that colloids are more efficient than crystalloids in reaching hemodynamic goals [52]. However, colloids are significantly more expensive than crystalloids, and there are no large-scale studies convincingly demonstrating a beneficial effect of colloids on patient-centric outcomes. It may not be fair to generalize amongst colloids, where the type of colloid also impacts outcome. The most straightforward example is hydroxyethyl starches, which are associated with a higher degree of renal injury and possible risk of death, without any clear benefits and, are therefore, not recommended for use in sepsis [1, 53].

There is a robust body of evidence evaluating the effects of albumin administration in critical illness in general and, more specifically, in sepsis and septic shock, either as a primary comparison or as a subset analysis of a larger trial. This has resulted in a suggestion from SSC to use albumin in addition to crystalloids for initial resuscitation and subsequent intravascular volume replacement in patients with sepsis and septic shock when patients require substantial amounts of crystalloids [42]. This recommendation is largely based on (1) the ALBIOS trial which reported albumin may be associated with decreased mortality in the subgroup of patients with septic shock and hypoalbuminemia [54] and (2) the SAFE trial which showed no benefit to albumin in overall survival in critical illness but a decreased mortality in the subgroup of patients with sepsis [55]. Importantly, in the ALBIOS trial, albumin was administered in hypoalbuminemic septic patients in an attempt to maintain a serum albumin level of 30 g per liter or more. In addition, albumin was given as a daily dose (up to 300 mL of 20% albumin) for 7 days. In contrast, a recent single-center trial of 360 septic patients with cancer failed to show beneficial effects for the addition of albumin on 30-day mortality [56]. Notably, studies comparing colloids with crystalloids used saline as a comparator. If buffered solutions are indeed superior to saline, the potential benefits of colloids reported in previous trials may have been related to disadvantages related to the saline comparator and should be investigated further, perhaps in the setting of a three-armed trial with a saline arm, a buffered solutions arm, and an albumin arm. Studies evaluating albumin primary test albumin as a maintenance fluid rather than a resuscitation fluid in the stages of initial resuscitation, primarily after crystalloids, have proved ineffective. There may be a role to evaluate albumin as a supplemental resuscitation fluid following initial resuscitation with crystalloids. Finally, there are no large-scale studies evaluating albumin as a resuscitation fluid, triggered by hemodynamic endpoints.

#### What is not known—gaps in our understanding—directions for future research

1. Are there individual patient populations in which saline should be avoided entirely? Testing types of fluids in subgroups of patients to detect the likelihood of adverse effects would be useful.
2. Are there differences between buffered solutions? Is lactated Ringer’s superior to acetated/gluconated buffered solution or vice versa?
3. What are the implications of acetate and gluconate contained in some balanced solutions? In cardiac surgery patients, an acetate-based balanced solution had similar hemodynamic effects compared with lactated Ringer’s [57]. However, significant infusion of acetate may contribute to vasoplegia and myocardial dysfunction in patients with kidney dysfunction [58, 59].
4. Recent data suggest that maintenance fluids and fluids used in diluting drugs may provide significant amount of chloride and have a major impact on fluid balance. Data suggest that selecting sodium/chloride poor maintenance fluids may improve fluid balance and chloride load in postoperative patients [60], but data in septic patients are still lacking. As such, should chloride containing solutions (including balanced solutions) be minimized or entirely avoided? Notably, this question is especially pertinent given recent instances of supply chain interruption, resulting in shortages of critical fluids and necessitating therapeutic substitutions at the individual patient level [61].
5. Are the beneficial renal outcomes associated with buffered solutions compared with saline due to the reduction in chloride content? In other words, is chloride toxic in itself, so that toxicity occurs even in absence of obvious hyperchloremia/acidosis (in which condition chloride-free solutions should be favored and saline-based dilution of drugs should be avoided whenever possible) or is it deviation of chloride/pH that generates the toxicity of high volumes of NaCl 0.9% (in which context closer monitoring of these variables may limit the toxicity)? Can lactated sodium solutions serve as a substitute for saline to minimize renal injury?
6. Is albumin superior to buffered crystalloid solutions? If used, when should albumin be administered? Ideally, this comparison of albumin with balanced crystalloid solutions would include subgroups of patients of particular interest (chronic kidney disease, cirrhosis).
7. Should albumin be initiated in an attempt to correct hypoalbuminemia or as a volume replacement fluid? Given the colloidal qualities of albumin, would albumin be of greater benefit in cases of severe shock, necessitating greater amounts of fluid resuscitation? Or should it be used to correct hypoalbuminemia? If there is a benefit to albumin administration as a fluid resuscitation therapy, where does it provide the greatest benefit in light of its cost?
8. What is the optimal concentration of the albumin solution (4–5% or 20%)? Studies of albumin have evaluated various concentrations of albumin. Contingent on its proposed mechanism of benefit in fluid resuscitation (correction of hypoalbuminemia vs intravascular fluid expansion), studies comparing the various albumin concentrations are necessary.
9. Should severity of sepsis play a role in determining which fluid to use for resuscitation? In the continuum of sepsis and septic shock, would patients specifically with more severe forms of shock benefit from a combination approach of crystalloids and colloids to fluid resuscitation?
10. Does resource setting play a role in determining the efficacy of which fluid is chosen? Studies outlined above were performed in resource-intensive environments. Considering data demonstrating that outcomes to fluid resuscitation are disparate between resource-intensive environments and resource-limited environments, it is possible that the type of fluid might also impact outcomes, understanding that fluid choices will likely be significantly more limited in resource \-constrained locations and in the face of unpredictable supply.

### Question 3: what is the optimal approach to selection, dose titration, and escalation of vasopressor therapy?

#### What is known

Mean arterial pressure (MAP) is determined, in part, by signaling through pharmacologically distinct families of receptors. Vasopressor agents are currently available that work by altering signaling via catecholamines, vasopressin receptors, and the renin/angiotensin system.

Norepinephrine is recommended as a first-line vasopressor agent for the treatment of septic shock [62]. A systematic review and meta-analysis of 11 randomized trials reported that norepinephrine use resulted in lower mortality (RR, 0.89; 95% CI, 0.81–0.98) and decreased risk of arrhythmias (RR, 0.48; 95% CI, 0.40–0.58) compared with dopamine [63]. Survey studies of intensivists reflect preferences for norepinephrine as the first-line vasopressor agent, reflecting clinician agreement with guideline recommendations [64–66].

In studies of vasopressin as both an adjunctive treatment and as a first-line therapy, results have been mixed. Although vasopressin did not alter mortality when added to norepinephrine, in a finding contrary to an a priori hypothesis, a subset demonstrated a potential and unexpected survival benefit in patients with less severe septic shock (norepinephrine < 15 µg/min) [67]. However, a subsequent study failed to confirm any survival benefit with vasopressin therapy although a reduced need for renal replacement therapy compared with norepinephrine was noted [68]. A consistent norepinephrine-sparing effect has been observed with vasopressin in doses of 0.01–0.03 U/min, leading to its recommendation as an adjunctive therapy [62].

More recently, angiotensin II was compared with placebo in a randomized controlled trial of 321 patients with vasodilatory shock requiring greater than 0.2 µg/kg/min of norepinephrine [69]. Angiotensin II was effective in achieving the primary endpoint for the study by increasing the MAP by greater than 10 mm Hg or pressure greater than 75 mm Hg. However, no difference was observed in the secondary endpoint of survival. A subsequent subgroup analysis of this trial demonstrated improved survival in 105 patients requiring renal replacement therapy at the time of randomization to angiotensin II, although this finding needs to be further investigated in a prospective, randomized trial [70].

Although norepinephrine, vasopressin, and angiotensin II work through different pathways, there are multiple agents that can impact the sympathetic system via altering levels of alpha and beta selectivity. Depending on the clinical scenario, additional stimulation or alpha or beta receptors could have potentially beneficial or deleterious effects. In this context, epinephrine is suggested as an additive agent to raise MAP and reduces the dose of norepinephrine in cases of refractory shock [62]. Although it is effective in raising MAP, epinephrine is not recommended as a first-line agent since clinical trials demonstrate a lack of a mortality benefit [71, 72], supported further by a meta-analysis of vasopressor trials [73]. Of note, epinephrine may increase lactate production via its activity on skeletal beta-2 receptors, complicating the interpretation of lactate clearance and evaluating perfusion. Phenylephrine represents a complementary vasopressor that could be used. Given its selective alpha-1 agonist activity, the pharmacology of phenylephrine may appear attractive in a high output hypotensive state; however, this agent has the potential to cause splanchnic vasoconstriction [74]. Notably, in a recent analysis of the effects of a nationwide shortage of norepinephrine, phenylephrine was the most commonly substituted vasopressor and was associated with increased mortality [75]. Although correlation is not equivalent to causation, this is not supportive of earlier usage of phenylephrine. Further, dopamine works not only through dopamine receptors at lower doses but also signals through alpha receptors at higher doses. In a randomized trial comparing norepinephrine to dopamine, mortality outcomes were similar with both drugs; however, dopamine was associated with a higher incidence of arrhythmias, which limits its utility if other agents are available [76]. In addition, the rising cost of available vasopressors challenges their application in clinical care [77]. In an environment where there are concerns due to the rising cost of healthcare and an increasing emphasis on value, the cost of vasopressor drugs is under scrutiny in order to justify use.

When administering vasopressors, blood pressure targets have been evaluated in a number of different studies. The SEPSIS-SPAM trial found that there was no difference in mortality when comparing a MAP target of 65–70 mm Hg to a MAP target of 80–85 mm Hg [78]. Notably, increasing MAP goal in patients with chronic hypertension led to reduced need for renal replacement therapy. However, targeting a higher MAP was associated with a greater incidence of atrial fibrillation. A pooled analysis reported that targeting higher MAP targets is associated with higher mortality in patients treated with vasopressors for more than 6 h [79]. More recently, a randomized trial comparing permissive hypotension (MAP 60–65) compared with usual care (defined as at the discretion of the treating clinicians allowing a more personalized approach) demonstrated no difference in mortality with a trend toward improved mortality in the former group [80]. Of note, mean MAP was 66.7 mm Hg in the permissive hypotension group and 72.6 mm Hg in the usual care group. The 65 Trial sought to determine optimal MAP target in a cohort of patients greater than or equal to 65 years old by randomizing subjects to a strategy of permissive hypotension (MAP target of 60–65 mm Hg) or usual care in the ICU to determine a difference of all-cause mortality at 90 days [80]. Importantly, this was a trial in the ICU setting following adequate fluid resuscitation as assessed by treating clinicians. At the conclusion of the trial, there was no difference in mortality although a notable difference in the point estimate (–2.85%; 95% CI, –6.75 to 1.05; *p* = 0.15). In the subgroup of patients with chronic hypertension (*n* = 1,131), there was a statistically significant difference in favor of the permissive hypotension strategy (adjusted odds ratio, 0.67; 95% CI, 0.51–0.88; *p* = 0.047). Although the 65 Trial did not yield a conclusive result, it did reinforce the logic that treatment goals need to be personalized to individual patients and that a lower MAP target may be acceptable and counterintuitively may be beneficial in elderly patients with chronic hypertension at baseline.

#### What is not known—gaps in our understanding—directions for future research

1. Should vasopressor agent selection be personalized based on patient characteristics (e.g., home medicines and chronic disease conditions)? A decisional process to determine how to individualize vasopressor therapies does not currently exist. Identifying patient-specific factors affecting organ perfusion and vasopressor response in order to adjust perfusion targets is not commonly used in clinical practice [81]. There is recognition that underlying chronic disease states may affect organ perfusion, yet there is a lack of evidence as to how to guide vasopressor selection based on patient-specific factors in the clinical setting.
2. What should the starting dose of norepinephrine be?
3. Is norepinephrine always the appropriate choice as a first-line vasopressor in sepsis? The data surrounding this recommendation are relatively scant. Is there a potential benefit to use vasopressin as a first-line therapy in select patients with septic shock in the broader population or in a select group of patients (e.g., atrial fibrillation, chronic kidney disease)?
4. Which agent should be considered as second-line therapy, and does this change based upon patient characteristics?
5. What are appropriate thresholds for adding a second vasopressor agent? Trials comparing adjunctive vasopressor strategies have evaluated different endpoints. Whether adding adjunctive therapies to norepinephrine improves safety and survival is largely unknown. Meaningful clinical endpoints aside from survival may potentially include (but are not limited to) need for renal replacement therapy, new arrhythmias, heart failure, thromboembolic disease, digit necrosis, and quality of life. This is vital to understand since adding an adjunctive agent to reduce the dose of norepinephrine without improving survival or reducing adverse events would result in increased costs, possibly without improving patient-centric clinical outcomes.
6. When should patients receive fluid resuscitation versus vasopressor initiation versus both?
7. When should angiotensin II be initiated? Although the ATHOS-3 trial established the efficacy of angiotensin II as a vasopressor agent to raise blood pressure, important questions remain regarding angiotensin II’s role in clinical practice, primarily due to the lack of comparative data and potential safety concerns related to the higher rate of thromboembolism observed in clinical trials to date. Although an argument can be made for the benefits of multimodal vasopressor therapy, more outcome and comparative data are necessary both to determine if angiotensin II is clearly beneficial in subpopulations (such as potentially in patients requiring renal replacement therapy) or is potentially harmful when compared with a strategy that does not use this agent.
8. What is the role of phenylephrine, if any, in the management of sepsis?
9. What is the ideal target MAP in septic patients?
10. How do chronic comorbidities (hypertension, CKD), and baseline medications, impact both goal MAP and vasopressor response?
11. What is the cost effectiveness of different vasopressor strategies? Although cost of care has traditionally not been prioritized, it is a significant factor in the clinical environment where utility and efficiency are measured by stakeholders. Understanding that healthcare economics vary widely between countries, in an environment where healthcare costs are rapidly increasing, evaluations of vasopressors need to factor in costs.
12. How should vasopressor therapies be weaned?

## Summary

This report expands on clinical concepts outlined in “Fluid Resuscitation and Vasopressor Therapy”, previously identified as priorities by the SSC Research Committee [8, 9]. The authors aimed to provide clinicians and researchers with a detailed and informative summary of pressing questions that require investigation in the realms of fluid resuscitation strategies and vasopressor administration. Optimal approaches to fluid resuscitation and application of vasopressor therapies have evolved over the past decade as knowledge accumulates in the field, necessitating iterations to previous questions. In a domain of sepsis research as ripe as shock treatment, we hope that this review serves as a roadmap for future trials in the field of septic shock treatment.
